# Isolated Fallopian Tube Torsion Concomitant With an Adenomatoid Tumor Arising From the Fallopian Tube: A Case Report

**DOI:** 10.1155/crog/7233415

**Published:** 2026-04-23

**Authors:** Fumitaka Mukouyama, Hiroshi Ishikawa, Aki Kinoshita, Mariko Inoue, Yoshinori Itsuka, Yohei Hosokawa, Kenichi Harigaya, Jun-Ichiro Ikeda, Kaori Koga

**Affiliations:** ^1^ Department of Obstetrics and Gynecology, Reproductive Medicine, Graduate School of Medicine, Chiba University, 1-8-1 Inohana, Chuo-ku, Chiba, 260-8670, Japan, chiba-u.ac.jp; ^2^ Department of Obstetrics and Gynecology, Chiba Kaihin Municipal Hospital, 3-31-1 Isobe, Mihama-ku, Chiba, 261-0012, Japan; ^3^ Department of Pathology, Chiba Kaihin Municipal Hospital, 3-31-1 Isobe, Mihama-ku, Chiba, 261-0012, Japan

## Abstract

Isolated fallopian tube torsion (IFTT) is a rare condition in which the fallopian tube undergoes torsion without ovary involvement. The causes of IFTT vary widely, ranging from intrinsic abnormalities of the fallopian tube itself to extrinsic factors affecting the peritubal environment. Here, we report a case of IFTT concomitant with an adenomatoid tumor arising from the fallopian tube. A 49‐year‐old triparous patient presented with sudden‐onset lower abdominal pain. Imaging findings revealed a 3‐cm solid mass in the subserosal area of the left fallopian tube, and laparoscopy confirmed IFTT involving a dark red and solid mass. Histologically, an adenomatoid tumor was identified in the twisted fallopian tube. Adenomatoid tumors of the fallopian tube are typically small, asymptomatic, and identified incidentally during gynecologic surgeries or imaging; therefore, this tumor is an extremely rare cause of IFTT. Adenomatoid tumors in the fallopian tube, even those as small as 3 cm, can result in torsion.

## 1. Introduction

Isolated fallopian tube torsion (IFTT) is a gynecologic emergency in which the fallopian tube undergoes rotation or twisting without any ovarian involvement. The incidence of IFTT is reported to be approximately 1 in 1.5 million individuals, even rarer than adnexal torsion, including ovarian cyst torsion. IFTT most commonly presents with unilateral or bilateral lower quadrant/pelvic pain, nausea, and vomiting. While hydrosalpinx is the most common cause of IFTT, these symptoms are not specific to IFTT. Differentiating between IFTT and adnexal torsion based on imaging findings is difficult, and a definitive preoperative diagnosis of IFTT remains clinically challenging [1].

Adenomatoid tumors of the fallopian tube are rare mesothelial tumors, with approximately 50 cases reported to date [2]. Most reported cases have been asymptomatic, often observed as an incidental finding during gynecologic surgeries for other conditions or through imaging studies performed for unrelated diagnostic purposes. A few cases of symptomatic adenomatoid tumors of the fallopian tube have been reported [3–5], but there are no reported cases of IFTT or adnexal torsion resulting from an adenomatoid tumor of the fallopian tube.

Here, we report a case of a symptomatic adenomatoid tumor of the fallopian tube concomitant with torsion in a perimenopausal patient who was treated successfully via laparoscopic surgery. As with previous reports, a definitive preoperative diagnosis was challenging in this case. We present the preoperative imaging and laparoscopic findings, and the histopathological findings of the resected specimen. Written informed consent to publish the case report and related images was obtained from the patient at the time of laparoscopic surgery.

## 2. Case Presentation

**Figure 1:**
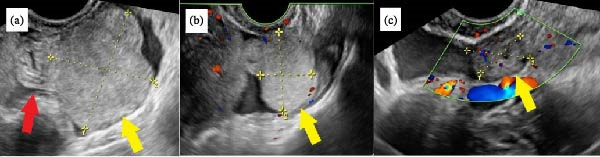
Diagnostic imaging findings. (a) Transvaginal ultrasound image showing a mass (yellow arrow) located in the subserosal area of the fallopian tube (red arrow); (b) color Doppler image showing no blood flow to the mass (yellow arrow); (c) color Doppler image showing reserved blood flow to the fallopian tube (yellow arrow).

**Figure 2:**
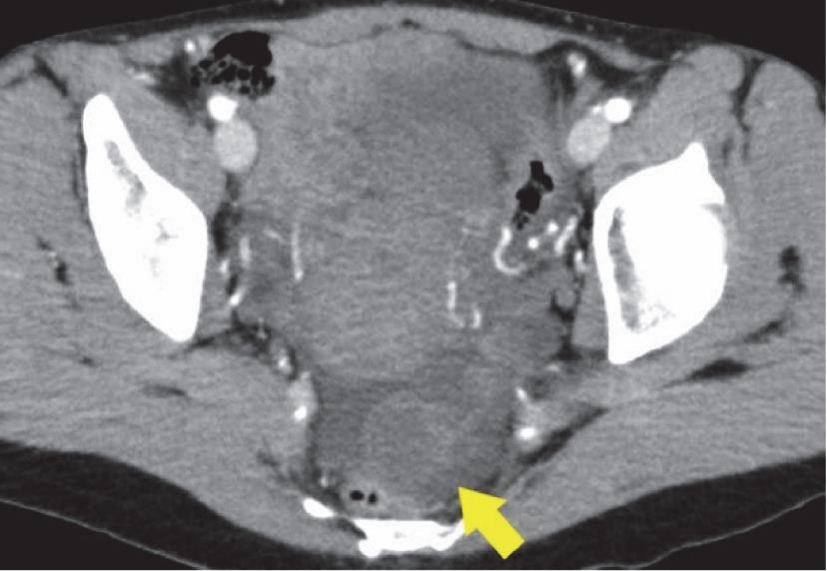
Diagnostic imaging findings. Computed tomography image showing the mass (yellow arrow) on the dorsal side of the uterus.

A 49‐year‐old gravida 3, para 3 woman without any relevant medical or surgical history presented to the emergency department with sudden onset, gradually progressing, persistent left‐sided lower abdominal pain. On palpation, the abdomen was soft and tender across the left lower portion. Transvaginal ultrasonography revealed a small mass, measuring 33 mm × 28 mm, in the diffusely edematous left fallopian tube (Figure 1a). The mass, located in the subserosal area of the fallopian tube, appeared as a well‐defined solid tumor with homogeneous isoechoic features. Color Doppler ultrasonography revealed no blood supply to the mass but a preserved supply to the fallopian tube (Figure 1b,c). Both ovaries were normal in size, with a small amount of free fluid observed in the rectouterine pouch. Routine blood test results indicated no significant findings: the white blood cell count and C‐reactive protein levels were within normal limits; the serum human chorionic gonadotropin test was negative; tumor markers for ovarian malignancies were within normal limits (cancer antigen 125, 23.9 unit/mL; cancer antigen 19–9, 6.0 unit/mL; and carcinoembryonic antigen, 1.2 ng/mL); and hormonal assays, including luteinizing hormone, follicle‐stimulating hormone, estradiol, and testosterone, were normal. Consistent with the ultrasound findings, contrast‐enhanced computed tomography (CT) revealed a 3‐cm solid mass on the dorsal side of the uterus that was considered part of the left adnexa (Figure 2); however, no apparent findings suggestive of torsion were observed. Based on these findings, differential diagnoses included paraovarian tumor, tubal neoplasm, pedunculated subserosal uterine fibroid, and adnexal torsion. Although imaging findings were not definitive, the acute onset of pain raised clinical suspicion for torsion.

**Figure 3:**
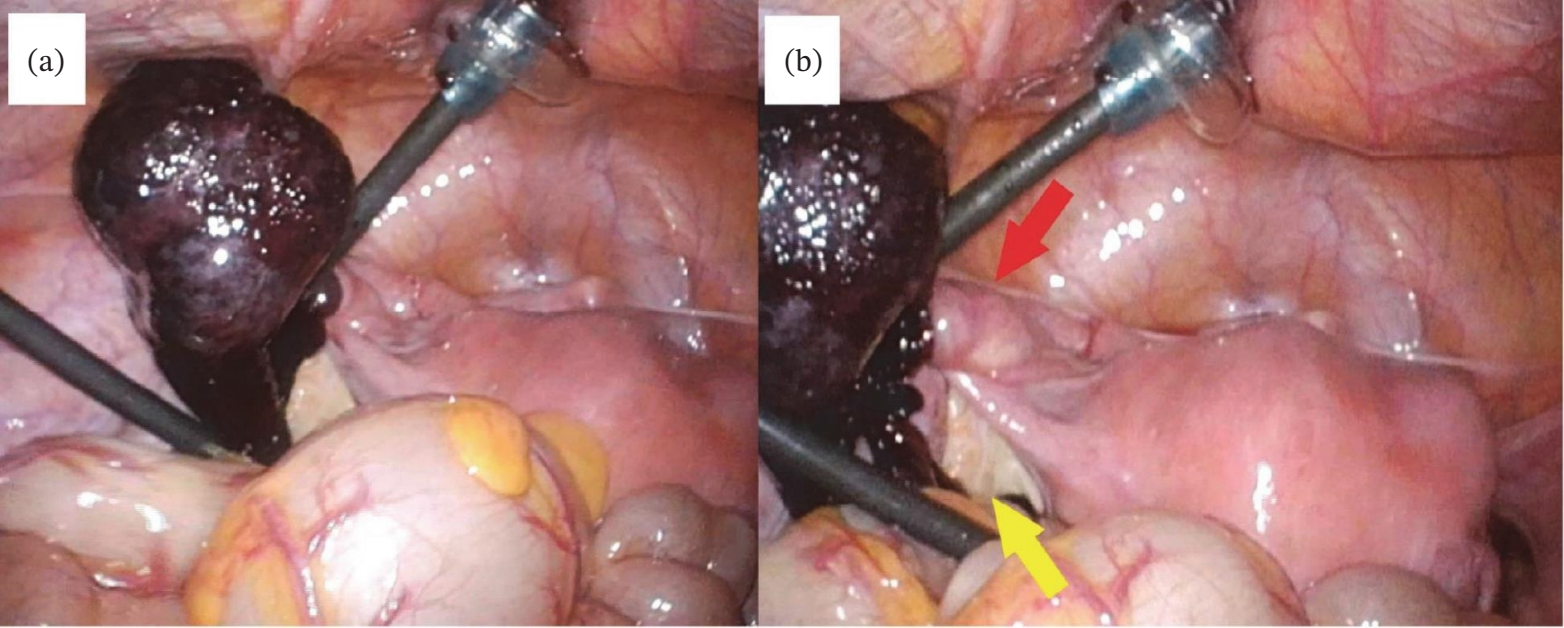
Laparoscopic findings of the twisted tumor. (a) The left fallopian tube along with the adenomatoid tumor with a congested, necrotic, and edematous appearance; (b) the yellow arrow indicates the left ovary of normal size, and the red arrow indicates the site of the fallopian tube torsion.

Laparoscopic surgery was performed after having obtained informed consent, which included the possibility of a second‐stage surgery if the tumor was found to be malignant. The laparoscopy showed that the twisted left fallopian tube was severely congested, hemorrhagic, and edematous (Figure 3). We also identified an approximately 3‐cm mass in the ampullary region of the torsed fallopian tube. The characteristics of the mass were unclear owing to severe congestion; however, the surface of the mass was smooth, and no adhesions to the surrounding tissues were observed, suggesting a benign condition. The left and right ovaries, fallopian tube, and uterus appeared normal, and neither adhesions nor abnormal tumors were observed within the pelvic cavity. We extracted 55 mL of hemoperitoneum and removed the left fallopian tube along with the mass following detorsion. The specimen, including the fallopian tube and the mass, was placed in an endoscopic retrieval bag and extracted through the umbilical trocar site. The laparoscopic surgery was completed in 1 h 30 min, with minimal intraoperative blood loss. The patient’s postoperative recovery was unremarkable.

**Figure 4:**
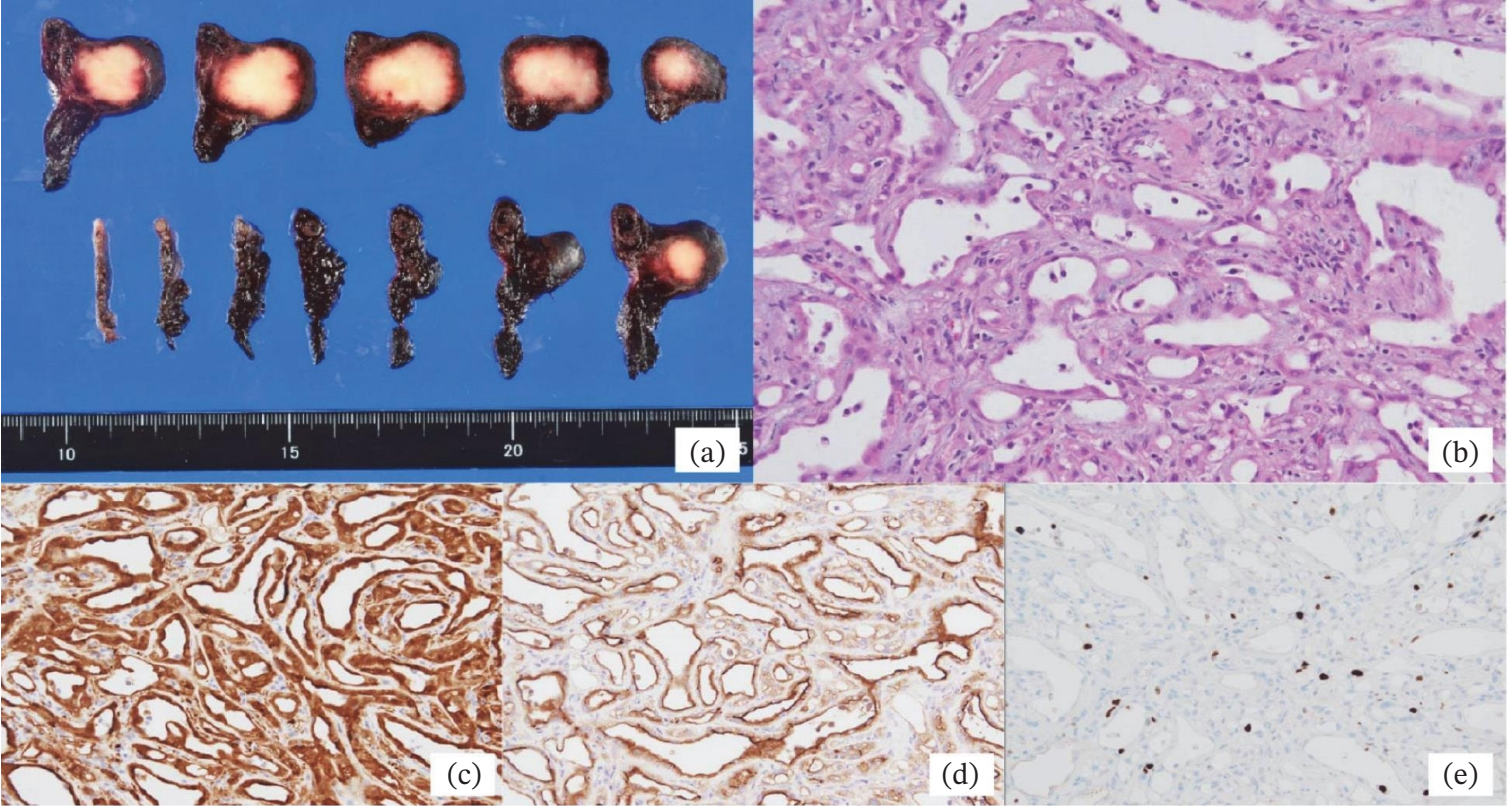
Histological findings of the fallopian tube and adenomatoid tumor. (a) Gross aspect of the tumor’s cut surface—firm with a grayish‐white cut surface; (b) hematoxylin and eosin staining, magnification × 200; immunohistochemical staining of (c) calretinin, (d) D2‐40, and (e) WT‐1 in the tumor, magnification × 200. The tumor exhibits a histological appearance typical of an adenomatoid tumor, with positive staining for mesothelial markers.

Histologically, the tumor measured 23 mm × 22 mm and was firm with a grayish‐white cut surface (Figure 4a). It originated from the subserosal region of the fallopian tube, extended into the subepithelial stroma, and was composed of tubular or cystic proliferations of flattened or cuboidal cells (Figure 4b). Immunohistochemical staining was positive for mesothelial markers, including calretinin, Wilms tumor 1 protein (WT‐1), and D2‐40 (Figure 4c–e). Among the epithelial markers, the mass was positive for cytokeratin AE1/AE3 and negative for Ber‐EP4. Estrogen and progesterone receptors and CD31, an endothelial marker, were negative. Cytological analysis of the ascites showed no malignant cells. These findings were consistent with those of an adenomatoid tumor, thereby confirming the diagnosis. No additional treatments were administered.

## 3. Discussion

IFTT is rarer than adnexal torsion, and its preoperative diagnosis is not always straightforward. IFTT can occur in various conditions, including pelvic inflammatory disease, hydrosalpinx or hematosalpinx, tubal neoplasms, ovarian and paratubal masses, trauma, and uterine masses, including a gravid uterus [6]. Hydrosalpinx is the most common cause of IFTT, occurring in 31% of patients [1]. Paraovarian cysts and paratubal cysts are reported to be another common cause of IFTT, with the risk of torsion increasing significantly when the cyst exceeds 5 cm in size [7]. A retrospective study of patients who underwent surgery for suspected adnexal torsion reported that the group diagnosed with IFTT had a higher prevalence of paratubal cysts detected on preoperative ultrasonography, presence of blood flow on Doppler imaging, and lack of torsion‐suggestive findings on preoperative CT or magnetic resonance imaging (MRI) than the adnexal torsion group [8]. Another retrospective study examining preoperative CT or MRI findings in patients surgically diagnosed with IFTT identified a characteristic pattern, which included the presence of a paraovarian cyst adjacent to a normal ovary, a thickened fallopian tube, and torsed vessels [9]. In the current case, preoperative CT revealed no definitive findings suggestive of IFTT, likely owing to the presence of ascitic fluid obscuring the condition of the adnexa and surrounding vessels; however, ultrasound findings, including a solid mass adjacent to a normal ovary and a thickened fallopian tube with preserved blood flow on Doppler imaging, are consistent with previously reported imaging patterns of IFTT.

Adenomatoid tumors, first described in 1945, are benign mesothelial neoplasms that predominantly originate from the genital tract, including the uterus, fallopian tubes, ovaries, testes, and epididymis [10, 11]. Adenomatoid tumors are more common in males than females, and adenomatoid tumors of the fallopian tube typically measure up to 1–2 cm and are largely asymptomatic [12, 13]. Most previous reports on adenomatoid tumors of the fallopian tube have focused on the clinicopathological characteristics of the tumors rather than the patient’s clinical courses. To our knowledge, this is the first case report in which an adenomatoid tumor of the fallopian tube was concomitant with IFTT or adnexal torsion. This case also highlights that even a small tumor can cause this condition.

Most adenomatoid tumors of the fallopian tube are an incidental finding during surgery for other gynecological conditions or unrelated medical imaging. In one reported case of an asymptomatic tubal mass identified during a routine gynecological examination, the tumor presented as a 20 mm multilocular solid mass on ultrasonography. The tumor was excised and subsequently diagnosed as an adenomatoid tumor [2]. However, a few case reports have suggested an association between adenomatoid tumors of the fallopian tube and symptomatic presentations such as ectopic pregnancy and chronic salpingitis [3–5]. Similar to our case, the presence of a tumor was not anticipated preoperatively in any of the reported cases. The current case presented as a well‐defined solid mass with homogeneous isoechoic properties, although these features might have been modified as a result of the torsion. Therefore, the preoperative diagnosis of adenomatoid tumors of the fallopian tube remains challenging owing to the lack of characteristic symptoms, combined with the absence of studies investigating the specific imaging features of adenomatoid tumors.

Adenomatoid tumors are macroscopically characterized with well‐defined, nodular, grayish‐white, firm‐cut surfaces [10]; histologically, they consist of flattened or cuboidal cells forming slit‐like, tubular, or cystic structures with thread‐like bridging within the luminal spaces [12–14]. Immunohistochemically, these tumors are positive for mesothelial markers such as calretinin, WT‐1, and D2‐40 [12, 13, 15]. The signet ring appearance of metastatic carcinoma involving the fallopian tube can mimic an adenomatoid tumor; however, the aforementioned markers are essential for obtaining an accurate diagnosis [16]. Among the epithelial markers, cytokeratins AE1/AE3 and CAM5.2 are largely positive in adenomatoid tumors [12, 13, 15], while Ber‐EP4 has been reported to be negative [15]. Adenomatoid tumors have been found to be negative for estrogen and progesterone receptors as well as CD31 [15]. In our case, the histological findings were consistent with those of a typical adenomatoid tumor.

In conclusion, we report a rare case of IFTT caused by a 3‐cm adenomatoid tumor arising from the fallopian tube, which was successfully treated with laparoscopic surgery. In this case, adnexal malignancy could not be entirely ruled out preoperatively; however, the patient was aware that a second‐stage surgery might be required if a malignancy was confirmed. Ultimately, the treatment was successfully completed using a single‐stage laparoscopic procedure, providing the patient with the most minimally invasive option. Further research is needed to help facilitate the preoperative diagnosis of adenomatoid tumors of the fallopian tube, given that adenomatoid tumors in the fallopian tube, even those as small as 3 cm, can result in torsion. Clinicians should be aware that even small adenomatoid tumors of the fallopian tube can cause torsion and should therefore be considered in the differential diagnosis of acute lower abdominal pain.

## Funding

This study was supported by a Health and Labour Sciences Research Grant from the Ministry of Health, Labour and Welfare of Japan (Grant 23FB1003).

## Consent

Written consent was obtained from the patient for the inclusion of personal information and images in the article.

## Conflicts of Interest

The authors declare no conflicts of interest.

## Data Availability

The data that support the findings of this study are openly available in ResearchGate at https://doi.org/10.13140/RG.2.2.11863.59049.
